# Severe Hyperlipidemia Induced Hemorrhagic Pancreatitis during Pregnancy

**DOI:** 10.1155/2009/383942

**Published:** 2008-01-28

**Authors:** Karen L. Koscica, Uzoma Nwaubani, Munir Nazir, Martin Gimovsky

**Affiliations:** Department of Obstetrics and Gynecology, Newark Beth Israel Medical Center, Newark, NJ 07712, USA

## Abstract

*Background*. We report a case of familial hyperlipidemia in pregnancy that resulted in hemorrhagic pancreatitis. *Case*. A patient at 27-week gestation was admitted for recurrent pancreatitis secondary to severe hyperlipidemia. With conservative care, the patient improved but on the fourth day of admission she experienced a sudden onset of hypotension and was diagnosed with hemorrhagic pancreatitis. *Conclusion*. Pancreatitis caused by hyperlipidemia is an uncommon event during pregnancy. A familiarity with the severe complications associated with this potentially life-threatening condition is important.

## 1. Introduction

Pancreatitis has been reported to occur in between one in 1000 to one in 12 000 deliveries. Gallstone disease accounts for most cases of pancreatitis during pregnancy. Shock and sepsis related to pancreatitis result in a relatively high mortality rate for both the mother and fetus. Most cases reported to date are mild in nature, although threatening hemorrhagic crisis may occur [1, 2]. We report on a patient with a history of familial hyperlipidemia complicated by acute hemorrhagic pancreatitis

## 2. Case

A 36-year-old gravida 6, para 3113 presented at 27-week gestation with abdominal pain. Her history was significant for acute pancreatitis secondary to familial hyperlipidemia during her first pregnancy. She presented in this pregnancy with the acute onset of left-sided abdominal pain and vomiting. On admission, her vital signs included a blood pressure of 110/70 and a pulse of 86. Her physical exam was positive for epigastric tenderness on palpation. Laboratory results revealed a hematocrit of 32.6% and an otherwise normal complete blood count. Her liver enzymes were elevated with an amylase of 149 U/L (normal 28–118 U/L) and lipase of 136 U/L (normal 6–51 U/L). She was admitted for conservative therapy with the diagnosis of pancreatitis based on her symptomatology, laboratory findings, and prior history. Her initial management consisted of intravenous hydration, bowel rest, and pain management. She was started on insulin due to elevated blood glucose values. A fasting lipid profile was performed because of her history of familial hyperlipidemia. Triglyceride level was greatly elevated 2944 mg/dl (normal <150 mg/dl).

**Figure 1:**
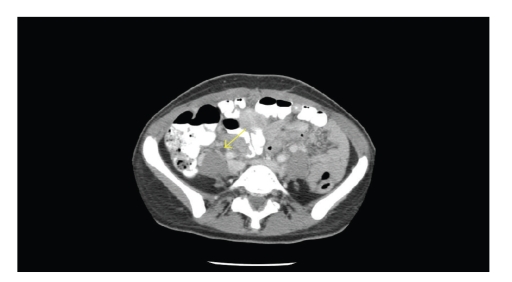
A CT scan of the abdomen showing hyperdense area adjacent to the pancreas and consistent with hemorrhagic pancreatitis.

By hospital day 4 her clinical condition had improved with a marked decrease in pain and vomiting. The amylase and lipase were persistently elevated at 265 U/L and 218 U/L, respectively. Later that afternoon, she experienced the sudden onset of dizziness, lightheadedness, and tachycardia. Her hematocrit decreased to 18.6%. On physical exam, there was no abdominal distension or flank bruising. Her vital signs revealed a blood pressure of 90/60 and a pulse of 110. Because of the precipitous drop in hematocrit, she was transferred to the intensive care unit. Consults with gastroenterology and surgery were obtained. A CT scan of the abdomen revealed multiple foci of hyperdense collections adjacent to the pancreas which were interpreted as consistent with hemorrhagic pancreatitis (see Figure 1). After a transfusion of 4 units of packed red blood cells, her hematocrit stabilized at 30%. She was placed on broad spectrum antibiotics and total parenteral nutrition. On hospital day 10, she became febrile and further abnormalities in her liver function tests were noted. Ultrasound and magnetic resonance cholangiopancreatography were performed and revealed no billiary obstruction or psuedocyst formation. Subsequently, the patient then went into preterm labor, and delivery was performed due to obstetrical indications. The fetus was in a breech presentation, and the patient desired repeat cesarean delivery. Tocolysis was not indicated because the patient had received previously a course of antenatal corticosteroids. A live infant with APGAR 8 and 8 and weighing at 1644 grams was delivered by cesarean section. Upper abdominal findings during surgery showed an indurated pancreas and a dilated gall bladder with no stones palpated. During her postoperative course, she was diagnosed with sepsis due to a Candida infection associated with the PICC line. Her jaundice and fever resolved, and she was discharged on postoperative day 9 after tolerating a low-fat diet.

## 3. Comment

Gallstone pancreatitis is still the most common form of pancreatitis seen during pregnancy. Other disorders that are associated with pancreatitis during pregnancy include alcohol abuse, trauma, viral infections, and medications. Hyperlipidemia is responsible for only a small percentage of cases that occur during pregnancy [1, 3]. Pregnant women with pre-existing hyperlipidemia are at higher risk for pancreatitis due to plasma triglyceride levels that increase between 2 to 4 folds during pregnancy in addition to a 50% increase in serum cholesterol. These changes have been ascribed to the increased estrogen levels present [1, 4]. As in our patient, triglyceride levels greater than 2000 mg/dl have been reported [1, 3]. A recent PubMed search using key words pregnancy, hemorrhagic pancreatitis, and hyperlipidemia revealed only 3 published cases.

The maternal mortality has been reported to be as high as 37% by Wilkinson in 1973 but a more recent study performed at a large public hospital in Dallas, Tex, USA reported no maternal deaths in 43 cases of pancreatitis during pregnancy [2, 5]. The perinatal mortality seen with acute pancreatitis in pregnancy is substantial and has been estimated at 20%. Most of the adverse perinatal outcomes are related to prematurity. Termination of pregnancy based on maternal status is rarely indicated except on a case-by-case basis, as in this case with acute hemorrhage and recurrence.

Complications of pancreatitis include psuedocyst, hemorrhage, infection, necrosis, shock, and maternal death. As in our patient, hemorrhagic pancreatitis is a potentially lethal condition in which the bed of the pancreas undergoes necrosis and becomes predisposed to bleeding. Blood loss may be severe and may be associated with a consumptive coagulopathy.

There is no clear consensus concerning management of pancreatitis. The mainstay of therapy for pancreatitis includes intravenous hydration, bowel rest, antimicrobials, and pain management. Surgery is reserved for recurrent biliary colic pancreatitis or for the patient who is not responding to conservative measures [2]. Surgery may also be chosen if the patient demonstrates any of the severe complications associated with acute pancreatitis, including necrosis or hemorrhage. Our patient was stabilized and recovered with aggressive product replacement and did not ultimately require surgical intervention.

Several medical therapies have been described for women with pancreatitis. Heparin has been used to lower triglyceride levels. There are reports in the literature in which heparin therapy for pancreatitis has been seen to be both therapeutic and safe [4, 6]. Although heparin is an option to control triglyceride levels, women with acute pancreatitis are at risk for life threatening hemorrhage within the pancreas, and therefore heparin could conceivably worsen the ultimate outcome. Additionally lipid lowering medication or plasma exchange has also been described in the literature as alternative therapies [1, 3, 6].

It is important for physicians who care for pregnant women not only to recognize acute pancreatitis but also appreciate its complications. Early recognition and prompt treatment may be life saving.
